# Measurement of Separase Proteolytic Activity in Single Living Cells by a Fluorogenic Flow Cytometry Assay

**DOI:** 10.1371/journal.pone.0133769

**Published:** 2015-08-12

**Authors:** Wiltrud Haaß, Helga Kleiner, Martin C. Müller, Wolf-Karsten Hofmann, Alice Fabarius, Wolfgang Seifarth

**Affiliations:** III. Medizinische Universitätsklinik, (Hämatologie und Onkologie), Medizinische Fakultät Mannheim der Universität Heidelberg, Mannheim, Germany; Virginia Tech, UNITED STATES

## Abstract

ESPL1/Separase, an endopeptidase, is required for centrosome duplication and separation of sister-chromatides in anaphase of mitosis. Overexpression and deregulated proteolytic activity of Separase as frequently observed in human cancers is associated with the occurrence of supernumerary centrosomes, chromosomal missegregation and aneuploidy. Recently, we have hypothesized that increased Separase proteolytic activity in a small subpopulation of tumor cells may serve as driver of tumor heterogeneity and clonal evolution in chronic myeloid leukemia (CML). Currently, there is no quantitative assay to measure Separase activity levels in single cells. Therefore, we have designed a flow cytometry-based assay that utilizes a Cy5- and rhodamine 110 (Rh110)-biconjugated Rad21 cleavage site peptide ([Cy5-D-R-E-I-M-R]_2_-Rh110) as smart probe and intracellular substrate for detection of Separase enzyme activity in living cells. As measured by Cy5 fluorescence the cellular uptake of the fluorogenic peptide was fast and reached saturation after 210 min of incubation in human histiocytic lymphoma U937 cells. Separase activity was recorded as the intensity of Rh110 fluorescence released after intracellular peptide cleavage providing a linear signal gain within a 90–180 min time slot. Compared to conventional cell extract-based methods the flow cytometric assay delivers equivalent results but is more reliable, bypasses the problem of vague loading controls and unspecific proteolysis associated with whole cell extracts. Especially suited for the investigaton of blood- and bone marrow-derived hematopoietic cells the flow cytometric Separase assay allows generation of Separase activity profiles that tell about the number of Separase positive cells within a sample i.e. cells that currently progress through mitosis and about the range of intercellular variation in Separase activity levels within a cell population. The assay was used to quantify Separase proteolytic activity in leukemic cell lines and peripheral blood samples from leukemia patients.

## Introduction

Aneuploidy, the occurrence of cells with too many or too few chromosomes, is a common characteristic of all tumors. [1] As already proposed by the German zoologist Theodor Boveri over 100 years ago, it is now widely accepted that aneuploidy promotes tumor progression and concurs with increased rates of chromosome missegregation when compared to normal diploid cells. [2,3] This phenotype is called chromosomal instability (CIN) and results in extensive karyotypic heterogeneity within a cancer cell population. [4] The intratumor heterogeneity is a major obstacle for efficient diagnosis, prognosis and therapy of human malignancies as tumor subclones with distinct aneuploidies feature an extreme phenotypic plasticity and can evolve depending on the selective pressure of the cancer-specific environment. [5–9] One of the major paths to aneuploidy is chromosomal missegregation caused by multipolar mitotic spindle formation due to supernumerary centrosomes (= centrosome amplification). [10–12] Centrosome amplification, in particular, the accumulation of additional centrosomes (n>2), is frequently detected in solid and hematological human cancers and has already been found in pre-neoplastic lesions i.e. early stages of carcinogenesis. [13–15]

Separase, a cysteine endopeptidase, is a key player in chromosomal segregation during mitosis. It performs proteolytic cleavage of the cohesin protein Rad21 during the metaphase to anaphase transition. [16] The function of uncleaved cohesin is twofold: First, it accounts for sister chromatid cohesion during DNA replication assuring proper chromatid pairing and chromosomal fidelity. Second, as a “glue” protein cohesin connects mother and daughter centrioles, the perpendicular oriented core structures of centrosomes. Once activated prior anaphase onset, Separase cleaves first the centrosomal pool of cohesin thereby promoting centriole disengagement that is the licensing step for centriole duplication in the next S phase. Subsequently, chromosomal cohesin is cleaved enabling segregation of sister chromatids via the mitotic microtubule apparatus. [17–19] In non-malignant cells where centrosomal duplication is tightly coupled to the cell cycle, Separase is activated just once per cell cycle round–just before anaphase onset–for a short period. Multiple inhibitory mechanisms combining Securin binding, specific serine residue phosphorylation (pSer1126) by CyclinB1/Cdk1, autocatalytic cleavage, and PP2A-dependent stabilization of Separase-bound Securin work together to prevent unscheduled activation of intracellular Separase. [20–22]

Overexpression and unscheduled activation of Separase results in premature separation of chromatids, lagging chromosomes and anaphase bridges that hinder proper segregation of sister chromatids at anaphase. [23] Moreover, hyperactive Separase uncouples centrosome duplication from cell cycle and leads to centrosome amplification, defective mitotic spindles and aneuploidy. [24] Numerous studies indicate that Separase is overexpressed and/or hyperactive in a wide range of human cancers and derived cell lines. [23,25–27] In a recently published study, Mukherjee and coworkers have demonstrated that Separase, when overexpressed in the mammary gland of a MMTV-Espl1 mice model, leads to the development of highly aneuploid mammary carcinomas with high levels of CIN and aggressive disease phenotypes. [28] Consequently, Separase has been identified as an aneuploidy promoter that, when overexpressed and hyperactive, functions as an oncogene and renders cells susceptible not only for chromosomal missegregation-induced aneuploidy but also for DNA damage and loss of key tumor suppressor gene loci associated with tumorigenesis and disease progression. [28,29]

Recently, Haaß and coworkers reported an increased Separase activity in CML patients undergoing long-term BCR-ABL-targeted treatment with tyrosine kinase inhibitors concurring with enhanced rates of acquired chromosomal aberrations (ACA). In consent with the before mentioned Darwinian model of clonal tumor evolution, they have hypothesized that therapy-triggered upregulation of Separase proteolytic activity in a small subpopulation of dormant leukemic stem/progenitor cells may play a role as promoting mechanism for the development of tumor heterogeneity, tumor progression and enhanced fidelity to escape therapeutic pressure. [27]

To date, there is no quantitative assay to measure Separase activity and its distribution on single cell level in leukemic cell populations. Current assays employ fluorogenic peptides that contain the Rad21 mitotic cleavage site as substrates and measure Separase activity in whole cell extracts after cell lysis. [26,30] In our hands, these assays worked quite well but were difficult to standardize due to vague assay loading controls. Moreover, depending on sample composition i.e. occurrence of cell types that express secretory proteinases (e.g. metalloproteinases, trypsin/chymotrypsin) as known from aggressive and highly invasive tumor cells, unspecific proteolytic activities may be associated with whole cell extracts and can distort measurement of Separase activity. [31,32] In whole cell extract-based assays the measured Separase activity levels are given as the mean of all cells lysed and analysis of distinct cell subpopulations is not possible without previous expensive and time-consuming cell sorting.

Here, we report the development and establishment of a flow cytometry-based Separase activity assay that allows detection and relative quantification of Separase proteolytic activity in single living cells. The assay utilizes a Cy5- and rhodamine 110 (Rh110)-biconjugated Rad21 cleavage site peptide ([Cy5-D-R-E-I-M-R]_2_-Rh110) as smart probe. Using a standardized protocol the FACS-based assay is a highly sensitive, specific and user-friendly tool that allows generation of tissue- and tumor-specific Separase activity profiles. Especially suited for the investigation of blood- and bone marrow-derived hematopoietic cells the assay was successfully used to monitor Separase proteolytic activity in leukemic cell lines and peripheral blood samples from leukemia patients.

## Materials and Methods

### Cell lines and culture conditions, nocodazole treatment

The human leukemic cell lines U937, MEG01 and BV173 were obtained from the DSMZ (German Collection of Microorganisms and Cell Cultures, Braunschweig, Germany). Cell line authentication was performed by DNA profiling commissioned at the DSMZ. All cells were cultured in RPMI-1640 medium supplemented with 10% fetal calf serum (FCS), 2% glutamine and 1% penicillin-streptomycin (Life Technologies GmbH, Darmstadt, Germany) (= complete RPMI-1640) at 37°C in 5% CO_2_ atmosphere. Exponentially growing cells were used in at least triplicate experiments.

For nocodazole treatment experiments 5x10^5^ cells/ml were propagated in complete RPMI-1640 medium containing 50 ng/ml nocodazole (# M1404, Sigma, St. Louis, MO, USA, stock 2 mg/ml in DMSO) for 20 h. To release the nocodazole block cells were washed twice and further incubated in fresh complete RPMI-1640 medium. After 0, 90, 180, and 270 min of incubation 1x10^6^/ml cells at a time were subjected to flow cytometric cell cycle and Separase activity analyses. In order to prevent an early release of U937 control cells (time point: 0 min) from nocodazole block the substrate incubation (90 min) was performed in the presence of nocodazole.

For serum supplementation experiments 3x10^5^ cells were propagated for 72 h without medium change. Then 1x10^6^ cells/ml at each case were fed with RPMI-1640 alone (0% FCS), with RPMI-1640 supplemented with 10% and 50% FCS, or with pure FCS (100%). After 2 h of incubation at 37°C in 5% CO_2_ atmosphere cell cycle and Separase activity were analysed.

### Patient and control samples

Ficollized peripheral blood mononuclear cells (PBMC) from three randomly chosen leukemia patients with CML (n = 3) in major molecular remission were investigated. At the time of blood sampling all patients have been receiving the tyrosine kinase inhibitors imatinib as maintenance therapy. Corresponding cells of healthy donors (n = 3) served as controls. Blood sampling was performed in the context of regular therapeutic monitoring. The procedure followed the declaration of Helsinki and was approved by the IRB/Medizinische Ethikkommision II der Medizinischen Fakultät Mannheim der Ruprecht-Karls-Universität Heidelberg. (http://www.umm.uni-heidelberg.de/inst/ethikkommission, # 2013-509N-MA from 2013-02-21). Written informed consent was obtained from all patients.

### Peptide synthesis

The rhodamine 110-labeled peptides [Cy5-D-R-E-I-M-R]_2_-Rh110, [Cy5-D-R-E-I-M-D]_2_-Rh110 and [Ac-D-R-E-I-M-R]_2_-Rh110 were synthesized by Biosyntan GmbH (Berlin, Germany). Instead of methionine (M) the analogous residue norleucine (Nle) was incorporated. All rhodamine 110-conjugated peptides were at least 95% HPLC pure. Stock solutions (1 mM) of fluorogenic peptides were prepared in DMSO.

### Flow cytometric Separase activity assay

Unless otherwise noted, 2x10^5^ cells were resuspended in 200 μl of complete RPMI-1640 medium containing a multiplex protease inhibitor cocktail (PI) selective for Serin-/Threonin proteases (Pefabloc SC, #76307 Sigma), Trypsin-/Chymotrypsin (#T9777, Sigma) and MMP-2/MMP-9 matrix metalloproteases (MMP-2/MMP-9 Inhibitor III, #444251 Calbiochem/Merck, Darmstadt, Germany). The final concentrations were 250 μM, 25 μM and 20μM, respectively. After 5 min of PI pre-incubation the fluorogenic peptide (10 μM final concentration) was added and the cells were incubated for 90 min at 37°C in 5% CO_2_ atmosphere. Fluorescence (10,000 signaling events in the viable population) of Rh110 (Ex_max_ 488 nm, Em_max_ 535 nm) and Cy5 (Ex_max_ 635 nm, Em_max_ 650 nm) was measured by fluorescence-activated cell sorting (FACS) using a flow cytometer FACSCalibur (Becton Dickinson, San José, USA). Discrimination of background fluorescence, cellular debris, cell doublets as well as gating of cells with peptide uptake (Cy5 positive cells) and cells displaying Separase activity (positive for Rh110 and Cy5) was performed by Kaluza software (Flow Cytometry Analysis Software, version 1.2, Beckman Coulter, Inc., Krefeld, Germany). FSC files were converted in text files by Flow Explorer 4.2 (Ron Hoebe, Amsterdam, The Netherlands) and imported in Microsoft Excel 2010 (Microsoft, Redmond, WA, USA) for further analysis. Peptide alone (Ac-D-R-E-I-M-R) without conjugated dyes (Cy5, Rh110) was used as non-fluorogenic control substrate and cellular autofluorescence gating control. For simultaneous cell cycle analysis living cells were stained with Hoechst 33342 (dilution 1/100,000, Sigma-Aldrich, Steinheim, Germany) and analyzed with a flow cytometer FACSCanto II (Becton Dickinson).

### Lysate-based Separase activity assay

About 60 μg cleared native protein lysate was analyzed in a quantitative fluorogenic assay according to Basu. [26] Spectrofluorometry was performed in 96 well Optiplate96F plates (Greiner-Bio-One, Frickenhausen, Germany) using a Tecan Infinite F200 Pro (Tecan GmbH, Crailsheim, Germany) at λex = 405 nm and λem = 465 nm.

### Separase silencing by espl1-directed siRNA

*Espl1*-specific siRNA (FlexiTube GeneSolution GS9700 for *espl1*) was purchased from Qiagen (Hilden, Germany). As negative control the same cells were transfected with AllStars Negative Control siRNA (Qiagen) a nonsilencing siRNA with no homology to any known mammalian gene. Transfection was accomplished using the Nucleofector manual (program T016, Lonza GmbH, Cologne, Germany). For siRNA treatment, 5x10^6^ U937 cells were resuspended in 100 μl Cell Line Nucleofector Solution (Lonza) containing 18 μl Supplement S Solution (Lonza). SiRNA was added to a final concentration of 0.01 nmol per 10^6^ cells.

### Cell cycle analysis

Subconfluent cells were harvested and washed in 1x phosphate buffered saline (PBS), subsequently fixed in icecold 75% ethanol and stained with propidium iodide (10 μg/ml propidium iodide, 2 mg/ml RNAse A in PBS). DNA content was measured by fluorescence-activated cell sorting (FACS) using a flow cytometer FACSCalibur (Becton Dickinson, San José, USA). Cell cycle analysis was performed with Flowing Software version 2.5.1 (by Perttu Terho, Turku, Finland).

### Western blot analysis, antibodies

Approximately 1x10^7^ cells per sample were incubated on ice for 20 min in 100 μl lysis buffer containing 50 mM Tris-HCl pH 7.4, 150 mM NaCl, 1 mM EDTA pH 8.0, 1% Triton X-100, 1 mM PMSF (Sigma-Aldrich), 2% complete protease inhibitor mix (Roche, Mannheim, Germany), 1% phosphatase inhibitor cocktails I and II (Sigma-Aldrich). Aliquots of clarified lysates were used for NanoQuant protein assays (ROTH, Karlsruhe, Germany). About 50–100 μg protein per lane were resolved by SDS-PAGE on BIORAD PreCast TGX 4–15% gradient gels, transferred to Immobilon-P membrane (Millipore, Bedford, USA) followed by blocking with 5% dry milk powder (AppliChem, Darmstadt, Germany) for 1h and immunostaining with the respective primary antibody dilution for 1 to 4h at RT or over night at 4°C. Primary antibodies (1:1000 dilutions): anti-CyclinB1 monoclonal mouse antibody (#MA1-46103; Pierce Biotechnology); anti-Securin monoclonal mouse antibody (#WH0009232M1; Sigma-Aldrich); Anti-Actin (13E5) rabbit mAb HRP Conjugate (#5125; Cell Signaling Technology, Leiden, The Netherlands) were diluted 1:10000. Signals were visualized with a ChemiDoc XRS+ System (BIO-RAD, München, Germany) after secondary antibody staining (goat anti-mouse IgG HRP conjugate antibody, goat anti-rabbit IgG HRP conjugate antibody (1:10000, Santa Cruz, Heidelberg, Germany) utilizing SuperSignalWest Femto Maximum Sensitivity Substrate (Thermo Fisher Scientific, Bonn, Germany). Image acquisition and densitometric analysis was performed using Image Lab Software (version 3.0.1, BIO-RAD). All values were normalized with Actin as loading control. Image cropping and tonal adjustments across the entire image were performed with Adobe Photoshop CS4 (Adobe Systems Inc., San Jose, CA, USA).

### Statistical analysis

All statistical calculations were done with GraphPad Prism software version 6.0 (GraphPad Inc., La Jolla, USA). Quantitative parameters are presented as mean values together with standard error of the mean (SEM). For qualitative data, absolute and relative frequencies are given. In order to compare two mean values, two-tailed unpaired Student’s tests were used. Test results with p<0.05 were considered significant. Values between p≥0.05 and p≤0.1 were defined as trend.

## Results and Discussion

### Peptide substrate design

**Fig 1:**
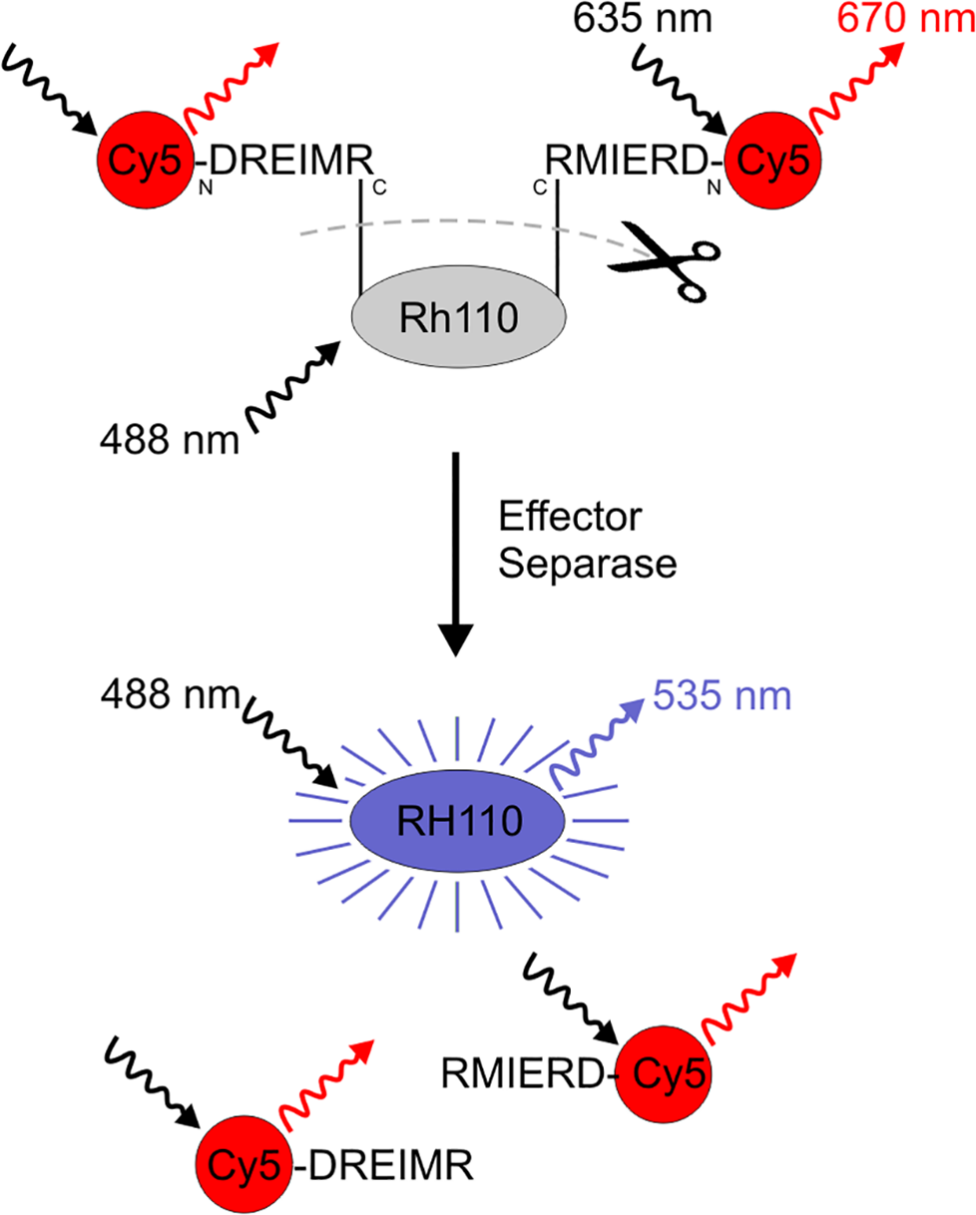
Schematic of fluorescent dye Rhodamine 110 (Rh110) activation following proteolytic cleavage by effector Separase. Rh110 contains two amino groups allowing for bi-conjugation to the C-terminal end of the Separase-specific substrate peptide ([Cy5-D-R-E-I-M-R]_2_-Rh110). When in this form, the molecule is essentially non-fluorescent. Active Separase cleaves the amide bonds between the peptides and the rhodamine. The Rh110 becomes fluorescently active and this increase in fluorescence can be measured by flow cytometry or fluorescence spectrophotometry (Ex_max_ 488 nm, Em_max_ 535 nm). Aminoterminal conjugation of Cy5 serves for monitoring peptide uptake by target cells as internal standard (Ex_max_ 635 nm, Em_max_ 650 nm). The location of amino- and carboxy-termini of the conjugated peptides are shown by the letters N and C, respectively.

We have established a cell-permeable fluorogenic peptide substrate for the detection and quantitative analysis of Separase proteolytic activity by flow cytometry in single living cells. The design is based on the previously described work of the Debananda Pati group who employed a 7-amido-4-methyl coumaric acid (AMC)- or a rhodamine 110-conjugated Rad21 peptide (D-R-E-I-M-R) that contains a Separase-cleaving consensus motif (E-X-X-R) as specific Separase substrate in whole cell extract-based activity assays. [26,30] Since the Rh110 fluorophore (Ex_max_ = 488 nm, Em_max_ = 535 nm) displays a much higher extinction coefficient, higher quantum yield and better photostability than AMC, we have used the latter ([Ac-D-R-E-I-M-R]_2_-Rh110; Ac = acetyl group) to further develop a dual fluorochrome, so-called “smart” probe suitable for use in flow cytometry. [30] As shown in Fig 1 the acetyl group was replaced by the fluorophore Cy5 (Ex_max_ = 635 nm, Em_max_ = 650 nm) resulting in the final dual fluorophore substrate [Cy5-D-R-E-I-M-R]_2_-Rh110 where two Cy5-conjugated peptides were linked to one Rh110 molecule. In this bi-conjugated form, Rh110 has very low autofluorescence due to the lactone state of the fluorophore. The Rh110-conjugated peptide mimics the Separase-recognition sequence. Once Separase becomes active, it cleaves the amide bond between the peptide and the rhodamine. The Rh110 becomes fluorescently active and this increase in fluorescence can then be measured. [30] The aminoterminal conjugation of Cy5 to each peptide molecule serves as internal standard as its fluorescence stays constant irrespective of peptide hydrolysis by Separase and is useful for monitoring substrate uptake in target cells. Furthermore, it prevents exoproteolytic degradation.

The use of synthetic fluorogenic peptide substrates for measuring enzyme activity in living cells joins in highly dynamic biochemical processes that are more or less beyond experimental control. Here, the Separase activity signal, i.e. the amount of Rh110 fluorescence generated by substrate cleavage, is a function not only of the number of intracellular Separase molecules and their proteolytic activities, but also depends on the kinetics of substrate delivery, substrate stability, cleavage specificity, Rh110 retention and adverse effects of unspecific peptidases potentially secreted by the cells of interest. In order to establish a robust, reliable and easy to use standardized FACS protocol we have addressed all mentioned parameters and have performed experiments on several human leukemic cell lines for optimization and validation of the experimental assay protocol.

### Substrate uptake and hydrolysis by Separase

**Fig 2:**
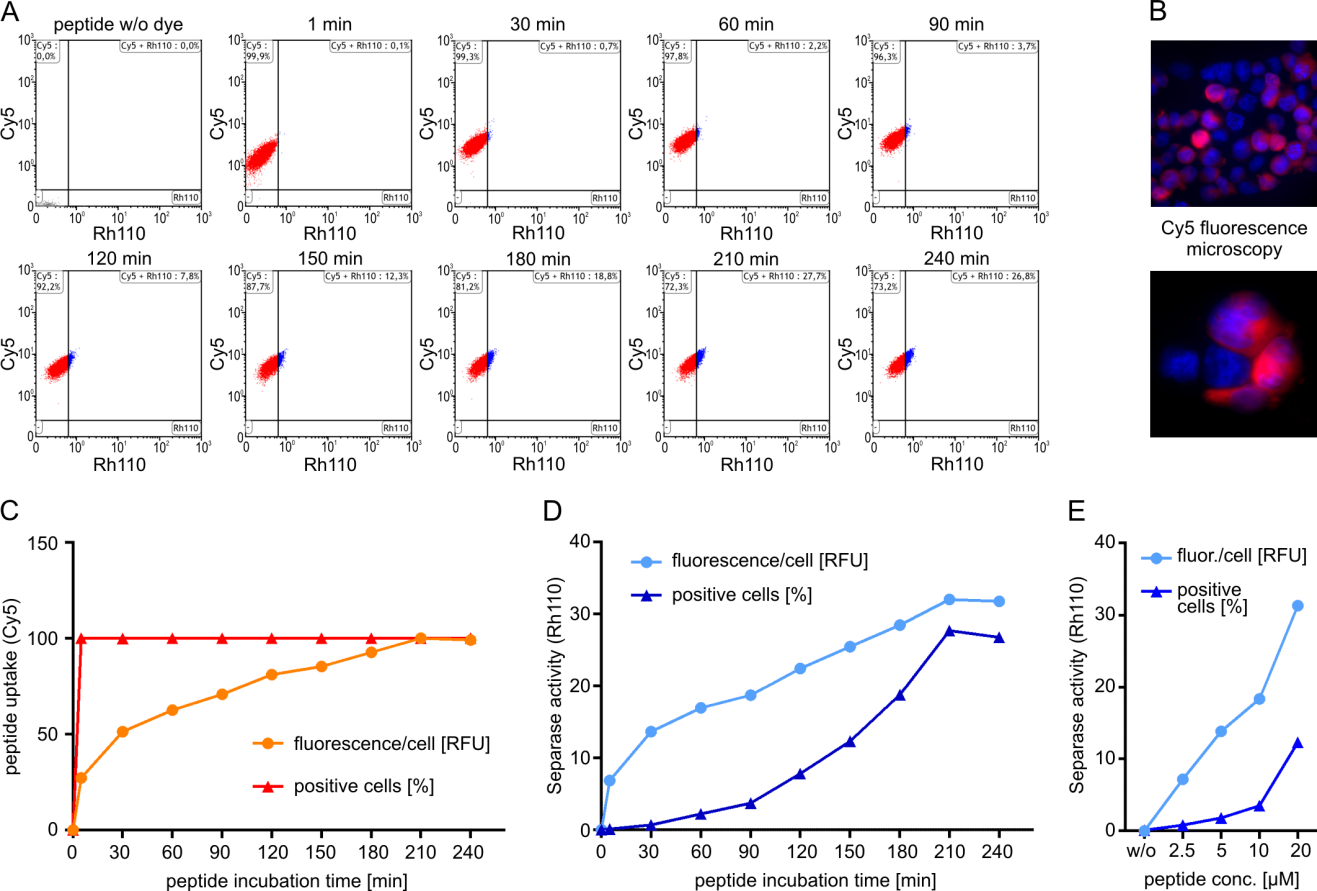
Kinetics and flow cytometric detection of [Cy5-D-R-E-I-M-R]_2_-Rh110 peptide uptake (Cy5 fluorescence) and peptide cleavage (Rh110 fluorescence) in U937 cells. (A) U937 cells were incubated with 10 μM peptide in complete RPMI-1640 medium for increasing time periods (1–240 min) followed by flow cytometric analysis according to the standard protocol as described in Materials & Methods. (B) Visualization of peptide uptake by Cy5 fluorescence microscopy. Equal amounts of peptide-treated (10μM, 90 min) and untreated cells were mixed and subjected to fluorescence microscopic analysis. Cy5 fluorescence is shown in pink, nuclei are counterstained with DAPI (blue). (C) Time kinetics of peptide uptake was calculated as the percentage of peptide (Cy5) positive cells (triangular dots) and the mean fluorescence per cell (round dots). (D) Time kinetics of Separase-related peptide cleavage was calculated as the percentage of Rh110 positive cells (triangular dots) and the mean fluorescence per cell (round dots). (E) Peptide dose (0–20 μM) dependency of Separase-related peptide cleavage. In all experiments, peptide alone (Ac-D-R-E-I-M-R) without dye (Cy5, Rh110) conjugation was used as non-fluorogenic control substrate and cellular autofluorescence gating control. Abbreviations: RFU, relative fluorescence units; fluor., fluorescence; conc., concentration; min, minutes.

To investigate the kinetics of [Cy5-D-R-E-I-M-R]_2_-Rh110 uptake into target cells, we incubated human leukemic U937 cells with 10 μM peptide substrate for increasing time periods (1–240 min) at 37°C followed by flow cytometric analysis. Since the addition of synthetic peptides may influence cellular basal autofluorescence the same peptide without dye (Ac-D-R-E-I-M-R) was used for unstained gating controls. As shown in Fig 2A–2C, substrate uptake in U937 cells was fast. Cy5 fluorescence was detectable in all cells after 1 min of incubation (the shortest time technically feasible). When estimating the relative fluorescence (RFU) per cell, the continuing substrate uptake reveals an increase of intracellular substrate levels approaching saturation after 210 min of incubation. Fluorescence microscopy points to cytoplasmic localization of the peptide substrate (Fig 2B). Within the tested time frame (1–240 min) of incubation no toxic effects were observed. Separase activity was recorded as the amount of Rh110 fluorescence released by intracellular substrate hydrolysis and became detectable after 30 min of incubation. Prolonged incubation resulted in a linear Rh110 signal gain (slope Δ_RFU_/Δt) within the 90–180 min time slot (Fig 2D) indicating a constant substrate conversion rate in U937 cells. The intracellular substrate concentration was not a limiting parameter. The number of Separase positive cells within this time slot ranged between 4 and 18% reasonably matching the proportion of U937 cells in G2/M phase (data not shown). Therefore, we considered an incubation time between 90 and 180 min best suited for the assay. In all following experiments described here 90 min of incubation time were applied. Varying substrate concentrations (2.5 to 20 μM) revealed a clear substrate/signal dose dependency pointing to a concentration-dependent uptake and hydrolysis of substrate within target cells (Fig 2E). The steady uptake of fresh substrate, the continuous cleavage of delivered substrate, the intracellular accumulation of released Rh110, but also cell cycle progress during incubation makes the assay highly dynamic and dependent on incubation time and substrate concentration. Therefore, for sample comparison the use of a standardized algorithm is crucial.

### Specificity of substrate cleavage

Separase substrate specificity (peptide D-R-E-I-M-R) has been thoroughly validated in several experiments by Pati and coworkers. [26,30] Using recombinant Separase purified by immunoprecipitation and activated by Ca^2+^ induced Securin degradation it has been demonstrated that caspase-related substrates are not cleaved by Separase pointing to the high selectivity of Separase proteolytic activity. [26] Furthermore, intracellular Separase-related proteases such as caspases-3/-7, the major effectors of apoptosis, are not able to cleave the ([Ac-D-R-E-I-M-R]_2_-Rh110) substrate. [30] Further confirmative data have been obtained in RNAi experiments where Separase expression has been knocked down in 293T cells. The resulting reduction in Separase protein levels led to decreased ([Ac-D-R-E-I-M-R]_2_-Rh110) substrate cleavage in Separase assays. However, Basu and coworkers have not completely excluded the possibility that besides Separase other proteases in whole cell extracts might be able to cleave the peptide substrate. [26]

**Fig 3:**
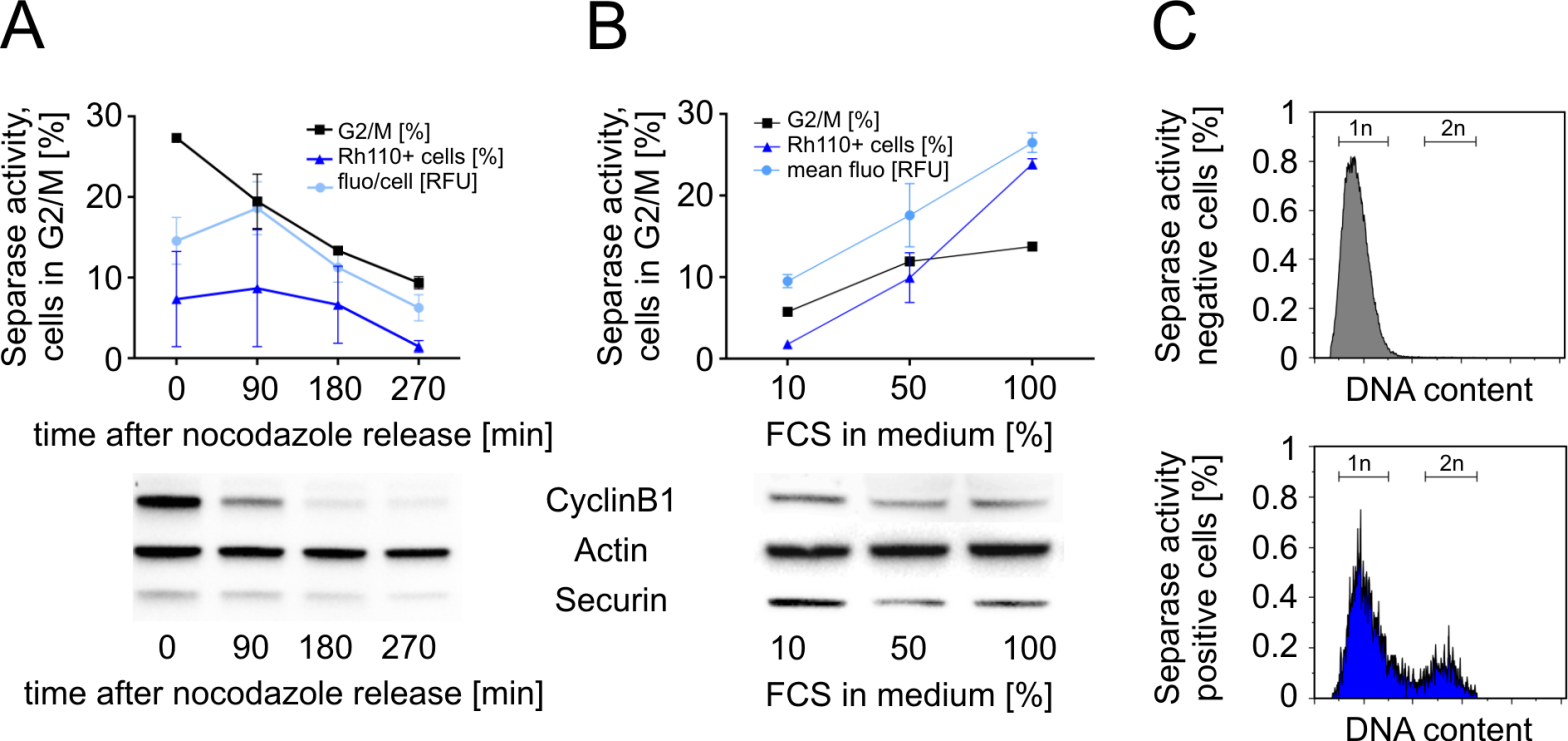
Separase activity during mitotic progression in U937 cells. (A) U937 cells arrested in G2/M by nocodazole were monitored by flow cytometry at 0, 90, 180, and 270 min after release from the nocodazole block. (B) Influence of FCS supplementation on Separase activity and mitotic progression. After serum starvation cells were incubated with increasing percentages (10%, 50%, 100%) of FCS for 90 min before flow cytometric analysis. Potential extracellular FCS-born Separase-unrelated proteolytic activity was inhibited by a cocktail of peptidase inhibitors (PI_mix_) as measured by cell lysate-based assay. Separase proteolytic activity was monitored as released Rh110 fluorescence. Corresponding Western blot immunostaining experiments (below) illustrate the expression levels of main Separase regulatory proteins (i.e. CyclinB1, Securin) that reversely correlate with Separase activity. Actin served as loading control. Cell cycle profiles were analyzed by flow cytometry after propidium iodide staining. The percentage of cells in G2/M as a measure of mitotic progression is depicted by the square dotted line. All assays were performed at least in triplicates. (C) Comparative analysis of DNA content in Rh110-negative (upper panel) and positive (lower panel) U937 cells after Hoechst 33342 staining simultaneously performed within the Separase assay. Abbreviations: FCS, fetal calf serum; fluo/cell, fluorescence per cell; 1n, haploid cells; 2n, diploid cells.

In contrast to the cell extract-based assay our FACS assay is performed on living cells with intact intracellular architecture (i.e. biocompartments). Therefore, the risk of unspecific proteolysis by other erroneously activated intracellular proteases is low. Nevertheless, to confirm the functional usability of the Separase substrate ([Cy5-D-R-E-I-M-R]_2_-Rh110) in our flow cytometric assay and to demonstrate the positive correlation between metaphase-to-anaphase transition (= Separase activation) and Rh110 signal gain we analyzed the influence of nocodazole treatment (Fig 3A) and overall proliferation on Separase activity in U937 cells (Fig 3B). Furthermore, the DNA content (Hoechst 33342) was comparatively analyzed in Rh110-positive and negative U937 cells to strengthen the correlation between cell cycle stage (1n or 2n) and measured Separase proteolytic activity (Fig 3C).

To measure Separase activity during mitotic progression, nocodazole arrested U937 cells were monitored by flow cytometry at 0, 90, 180, and 270 min after release from the nocodazole block (Fig 3A). About 30% of U937 cells were found to be in G2/M state at the start of the nocodazole release (t = 0 min). About 90 min after release from the G2/M block a Separase activity peak was detectable pointing to joint metaphase-to-anaphase transition of the released cells. As expected, Separase activation coincided with degradation of master Separase activity regulators CyclinB1 and Securin (Fig 3A, lower panel). Their reverse correlation with Separase activity in corresponding Western blot immunostaining experiments confirms that the measured proteolytic activity is in fact associated with Separase activation in living U937 cells. [27]

Separase activity, i.e. the number of Separase-active cells, should also correlate with the proliferation rate of the cell culture since nutrient matter, particularly growth factors supplemented with addition of FCS, are limiting. To test the influence of FCS supplementation on Separase activity and mitotic progression, starved U937 cells were incubated with increasing percentages (10%, 50%, and 100%) of FCS for 90 min followed by flow cytometric analysis (Fig 3B). Increasing the FCS percentage from 10% to 100% doubled the proportion of G2/M cells (5% to 12%) and concurs with an about 12fold increase in Separase-positive cell number. The corresponding Western blot immunostaining experiments confirm again that the measured FCS-dependent proteolytic activity is associated with Separase activation (Fig 3B, lower panel). To exclude the possibility that FCS-born proteolytic activities interfere with measured Separase activity we have analyzed FCS samples alone in the cell extract-based activity assay. No competing proteolytic activities were detected (data not shown).

The comparative analysis of the DNA content in Rh110 positive (= Separase active cells) and Rh110-negative U937 cells (lacking Separase activity) revealed that nearly all Separase-negative cells (96.15%) displayed a haploid (1n) genome (Fig 3C). 3.85% of the analyzed U937 cells turned out positive for Separase proteolytic activity as to be expected (compare Fig 2A, incubation time 90 min). The observed Rh110 fluorescence in Separase-positive cells was allocated to diploid (2n, 20% of cells) and haploid (1n, 57%) cells. While Separase activity in diploid cells can be well explained by the occurrence of cells in anaphase, Separase activity in haploid cells is unexpected but may be explained as follows:

1. After cytokinesis the resulting daughter cells share half of the Rh110 fluorescence thereby producing an “afterglowing progeny” that may well explain the Rh110 fluorescence in the haploid cells fraction.
2. As Separase autocleavage and/or Securin and Cdk1/CyclinB1-related re-inhibition is generally used to explain the shut down of Separase, to our knowledge no experimental data are available about the fate of Separase molecules and their activities after mitotic exit. [16] It may be possible that Separase is still functional for a certain time in the progeny cells. This possibility is supported by the time activity kinetics of purified Separase reported by Basu and coworkers. [26] They observed cleavage activity of Separase up to 6 h in cell-free extracts at 37°C. Furthermore, during Separase activation free Securin molecules are degraded more rapidly than Separase-bound Securin both leading to an almost complete depletion of total Securin within 44 min after APC/C activation. [33] Due to this momentary lack of inhibitory Securin upon mitotic exit residual Separase molecules may retain proteolytic activity in haploid daughter cells during FACS assay incubation time (90 min). These conceivable events may also contribute to the observed Rh110 fluorescence in haploid cells.
3. Numerous reports underline the oncogenic character of Separase and state that its temporal and spatial regulation can be uncoupled from the cell cycle i.e. Separase activity may not be restricted any more to the mitotic anaphase. [24,29,34] Instead, a defective licensing of Separase-dependent centriole duplication may lead to iterative activation of Separase and may cause unscheduled rounds of centrosome replications contributing to DNA damage and karyotypic alterations commonly observed in human tumor cells. Since the majority of current cell culture models descent from tumor cells, we cannot exclude that the U937 cell line exhibiting defective centrosomes in 6% of cells (data not shown) displays low levels of cell cycle-uncoupled Separase proteolytic activity. This may also contribute to the appearence of Rh110 fluorescence in our U937 experiments irrespective to the cellular mitotic status.

**Fig 4:**
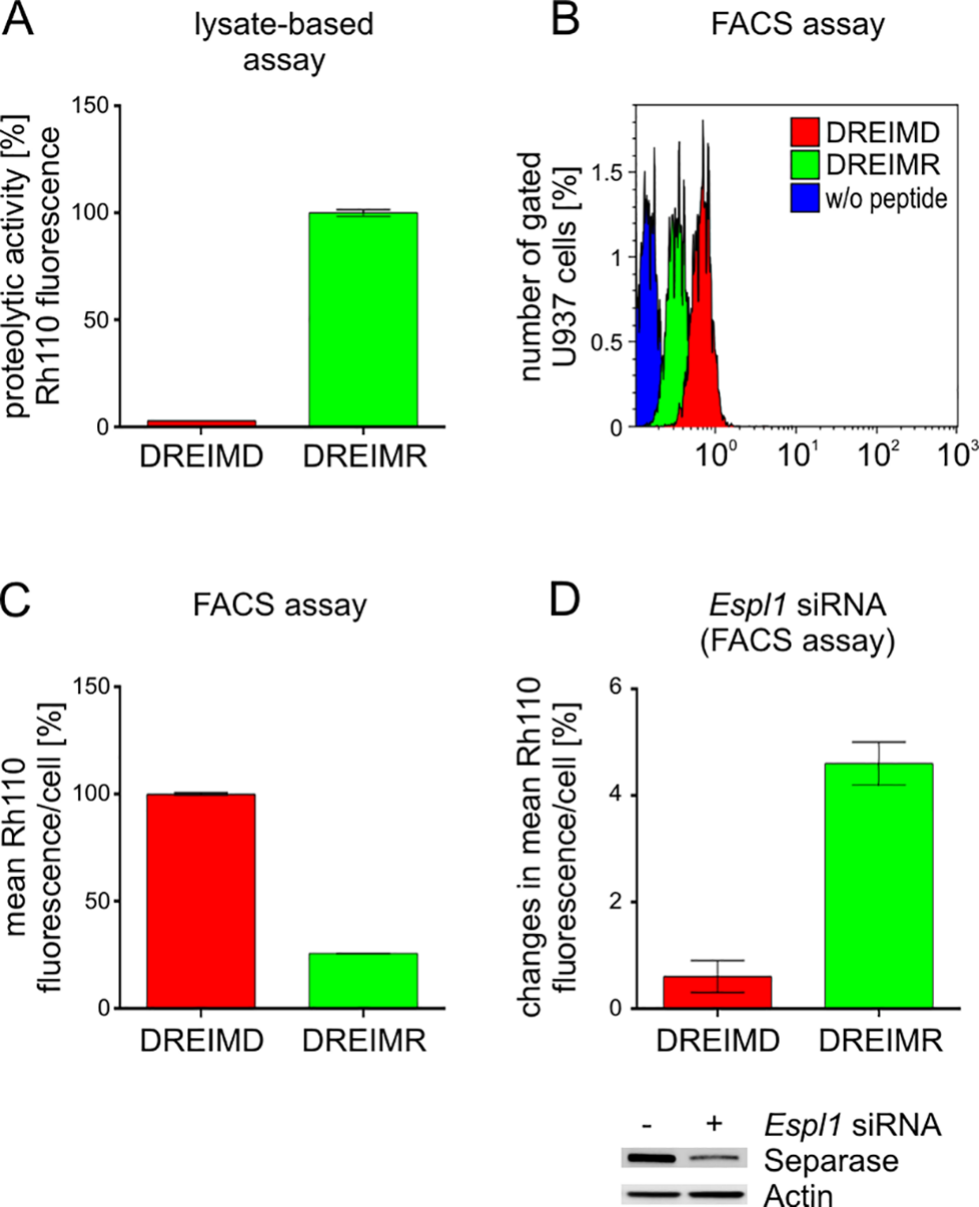
Comparative cleavage characteristics of the peptidic substrates ([Cy5-D-R-E-I-M-R]_2_-Rh110 and ([Cy5-D-R-E-I-M-D]_2_-Rh110 in U937 cells. The Separase-specific conjugated peptidic substrate (DREIMR) is shown in green, the control substrate lacking the Separase cleavage consensus (DREIMD) is depicted in red. (A) Substrate cleavage in the cell extract-based Separase assay as monitored by released Rh110 fluorescence. (B) FACS measurement of Rh110 positive cells after incubation with substrates according to the standardized protocol (90 min at 37°C). (C) Calculation of mean proteolytic activity per cell as measured by released Rh110 fluorescence in the FACS assay. (D) Changes (Δ-values) of proteolytic activities on substrates DREIMR and DREIMD after 48 h of *Espl1* silencing when compared to mock treated U937 cells. Corresponding Western blot immunostaining experiments (below) illustrate the knockdown of the Separase protein levels (siRNA (-), 100%; siRNA (+), 24%). Actin served as loading control. Abbreviations: DREIMD, ([Cy5-D-R-E-I-M-D]_2_-Rh110; DREIMR, ([Cy5-D-R-E-I-M-R]_2_-Rh110.

For a further characterization of the Separase substrate ([Cy5-D-R-E-I-M-R]_2_-Rh110) and its specificity, we performed analogous assays with a similar control substrate lacking the Separase-cleavage consensus ([Cy5-D-R-E-I-M-D]_2_-Rh110). In parallel, we knocked down Separase protein levels by siRNA-directed *Espl1* silencing (Fig 4). First, we compared cleavability of the Separase-specific substrate (conjugated peptide: DREIMR) with that of the control probe (conjugated peptide: DREIMD) using the cell extract-based assay (Fig 4A). Only minimal cleavage (2.8 ± 0.05%) compared to DREIMR (100 ± 1.5%) was observed for the control peptide pointing to the high specificity of the substrate ([Cy5-D-R-E-I-M-R]_2_-Rh110 in the cell extract-based Separase assay. Unexpectedly, when both peptide substrates were comparatively tested in the FACS assay, the control peptide did not behave inert but showed a much higher cleavage performance than the regular peptide DREIMR under our standardized assay conditions. This referred not only to the number of “Separase active “U937 cells (Fig 4B) but also to the mean Rh110 fluorescence per cell (Fig 4C). Control peptide cleavage was observed in 72.8% of all gated cells whereas DREIMR cleavage was detectable only in 3.8% of cells. The mean fluorescence in “Separase”-positive cells was 2.6fold higher in ([Cy5-D-R-E-I-M-D]_2_-Rh110 (100 ± 0.3%) than in ([Cy5-D-R-E-I-M-R]_2_-Rh110 (38.9 ± 0.3%) treated U937 cells. Since no differences in the cellular uptake of both peptides could be observed (data not shown), we assume that another so far unidentified intracellular protease efficiently cleaved the control substrate. This unknown protease seems to be inhibited in the extract-based assay by the added protease inhibitors that leave Separase active. In the FACS-based assay that deals with living cells and an intact intracellular architecture, the protease inhibitor cocktail added to our standardized incubation mixture was obviously not able to inhibit the intracellular protease. However, this protease seems not to cleave the DREIMR substrate because in this potential case, much higher signal intensities and incidences should have been expected. The knockdown of Separase protein levels (24% of the mock control) by *Espl1* silencing revealed an about 8fold (4.6 ± 0.4% vs. 0.6 ± 0.3%) increase of Rh110 signal intensity in U937 cells treated with the Separase-specific substrate when compared to cells treated with the control substrate (Fig 4D). This behaviour of Separase to posttranslationally raise its activity upon protein level decrease matches our previously published data generated with the cell extract-based assay. [27] There, we reported on a tumor-related feedback mechanism that posttranslationally stimulates Separase proteolytic activity after therapy-induced decreases in Separase protein levels. On the basis of the clearly distinct cleavage characteristics of the Rh110-conjugated DREIMR and DREIMD peptidic substrates we suggest that the Separase FACS assay is specific and functional. Nevertheless, we emphasize that we cannot completely exclude the possibility that other intracellular proteases might potentially contribute somewhat to the ([Cy5-D-R-E-I-M-R]_2_-Rh110 substrate cleavage as already acknowledged by Basu and coworkers. [26]

### Retention of signal (= released unconjugated Rh110)

Once released from the peptide substrate by Separase-related hydrolysis the retention of free Rh110 molecules within the cells to be analyzed is of highest relevance. Detectable fluorescence signals will only build up if the Separase-related delivery of free Rh110 predominates the efflux of unconjugated Rh110 from the cells. Previously, the intracellular accumulation, retention and cytotoxicity of unconjugated Rh110 has been thoroughly analyzed in mouse and human cells. [35] As shown by microspectrofluorometry, the rate of Rh110 clearance may be short as Rh110 elimination was complete within 30 min in human lymphoblastoid cells (CCRF-CEM). Cell growth and morphology have been reported unchanged in the presence of Rh110 up to concentrations of 100 μM. [35,36]

**Fig 5:**
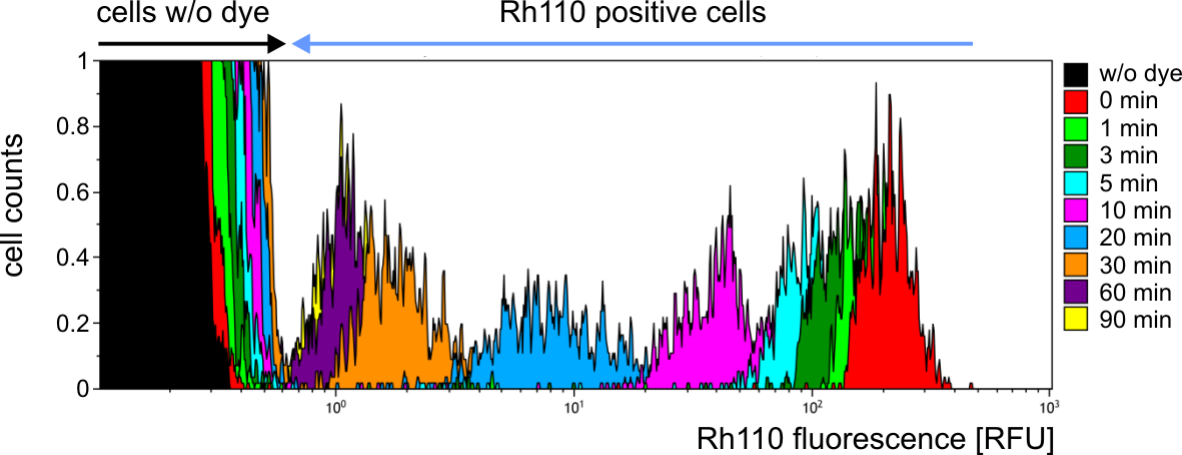
Efflux and influx dynamics of unconjugated Rh110. A mixed pool of U937 cells representing about 95% negative and 5% Rh110 positive cells (staining: 10 μM for 90 min) was incubated for 90 min while successively monitoring Rh110 fluorescence at given time points (0, 1, 3, 5, 10, 20, 30, 60, and 90 min). The time-related Rh110 fluxes from stained to unstained cells are visualized by colored histogram overlay. Abbreviations: RFU, relative fluorescence units; min, minutes.

To investigate the efflux and influx dynamics of unconjugated Rh110 within our assay a mixed pool of U937 cells representing about 95% Rh110-negative and 5% Rh110-positive cells (Rh110 concentration 10 μM) was incubated for 90 min while successively monitoring Rh110 fluorescence by FACS at distinct time-points (0, 1, 3, 5, 10, 20, 30, 60, and 90 min). The ratio 5:95 of stained to unstained cells was chosen to emulate real assay conditions (compare cell numbers at time-points 90 min and 120 min in Fig 2A). As shown in Fig 5, the time-related Rh110 fluxes from stained to unstained cells were visualized using a colored histogram overlay. Within 90 min of incubation a steady loss of Rh110 signal (about 2 logs in fluorescence) was observed in Rh110 pre-stained U937 cells while unstained cells gain slightly Rh110 fluorescence by the uptake of free Rh110 from cell culture medium. However, within the 90 min time slot discrimination between Rh110 stained and unstained U937 cells is still clearly possible. Moreover, in the real Separase assay the continuous delivery of cleavable substrate ([Cy5-D-R-E-I-M-R]_2_-Rh110) provides a steady increase in Rh110 fluorescence as long as intracellular Separase keeps active. Therefore, the inevitable signal loss by Rh110 leakage should be of inferior importance for discriminating Separase positive and negative cells in the FACS assay.

### Extracellular unspecific proteolytic activity

Extracellular unspecific proteinases may perform premature cleavage of substrate ([Cy5-D-R-E-I-M-R]_2_-Rh110) molecules in the incubation medium during the assay procedure. This is highly undesired as it can degrade considerable amounts of the supplemented peptide substrate (10 μM). Furthermore, it may lead to false positive signals or higher background fluorescence when unconjugated Rh110 enters and stains Separase-negative cells (as addressed in Fig 5).

Secretory proteases play essential roles in many physiological processes such as food digestion, blood coagulation, fibrinolysis, tissue remodeling, wound healing, cell proliferation, inflammation, tumor invasion and atherosclerosis. [32] Of these, metalloproteinases and serine proteinases constitute the major group and are commonly found in human plasma and white blood cells where they act on haemostasis and immune responses. [31]

**Fig 6:**
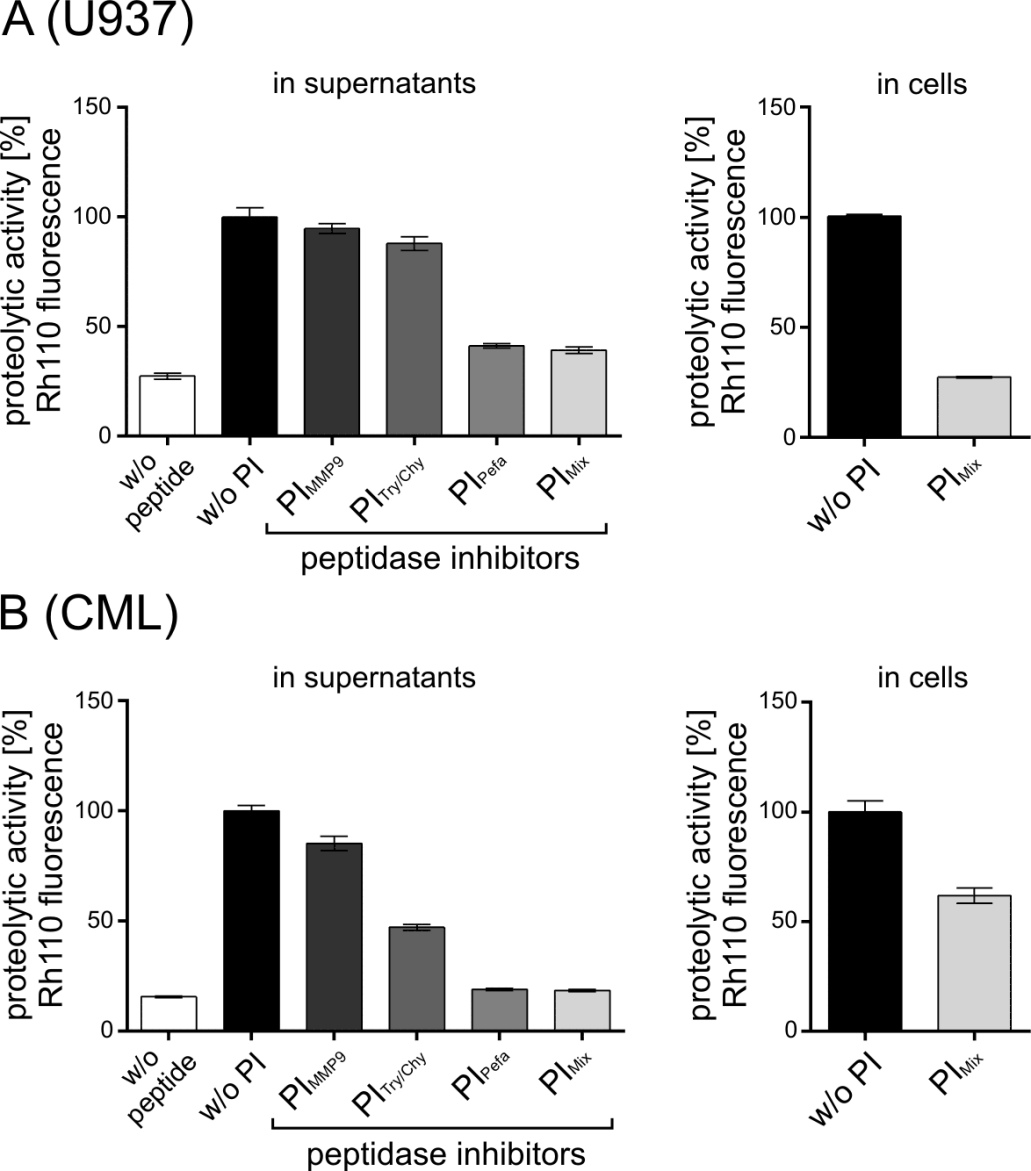
Influence of unspecific extracellular proteases on Separase assay results. Rh110 fluorescence in U937 cells (A) and in PBMC of a patient with chronic myeloid leukemia (B) as well as in the corresponding cell supernatants were assayed by flow cytometry and lysate-based assay, respectively. Three protease inhibitors alone (Pefabloc SC, soybean trypsin-chymotrypsin inhibitor, MMP-2/MMP-9 inhibitor III) or in combination (PI_mix_) were used to suppress extracellular Separase-unrelated proteolytic activities according to the recommendations of the manufacturers. Proteolytic activity was measured as released Rh110 fluorescence. Abbreviations: RPMI, RPMI-1640 cell culture medium; PI, protease inhibitor; MMP9, matrix metalloproteinase 9, Pefa, Pefabloc SC; Tryp/Chym, trypsin-chymotrypsin inhibitor; PI_mix_, cocktail of all combined peptidase inhibitors. All assays were performed at least in triplicates.

To analyze the occurrence and the potential influence of undesired extracellular proteolytic activities on Separase assay results and to test whether these interfering activities can be minimized by the use of adequate protease inhibitors we performed Separase assays employing both the whole cell extract-based assay according to [26] and the FACS-based assay. Three protease inhibitors alone (Pefabloc SC, soybean trypsin-chymotrypsin inhibitor, MMP-2/MMP-9 inhibitor III) or in combination (PI_mix_) were used to suppress extracellular non-Separase proteolytic activities in the supernatants of U937 cells (Fig 6A) and of PBMCs derived from a CML patient (Fig 6B). The measurement of Rh110 fluorescence levels revealed that all three inhibitors are able to reduce extracellular proteolytic activity. While the matrix metalloproteases MMP-2/-9 seem to contribute little to extracellular proteolysis (about 5 and 15%), serine proteases such as trypsin and chymotrypsin perform most of the inhibitable proteolytic activities as their inhibition revealed about 61% and 82% reduction in total proteolytic activity in U937 supernatant and CML plasma, respectively. A cocktail of all three inhibitors (= PI_mix_) resulted in almost complete inhibition of all competing extracellular peptidases. Therefore, PI_mix_ was considered as an essential ingredient of our standardized assay protocol and was used in all described experiments.

When PI_mix_ was applied in the FACS assay, about 73% and 38% of measured Rh110 fluorescence was lost in U937 and leukemic cells, respectively. It remains unclear whether this drop was due to intracellular inhibition of Separase, inhibition of a non-Separase peptidase or to the diminished uptake of extracellular unconjugated Rh110 that may be generated in supernatants omitting the PI_mix_. Although we cannot completely exclude the existence of small amounts of competing proteolytic activities we consider the remaining proteolytic activity as Separase-associated. This is in complete agreement with the results of our nocodazole experiment (Fig 3) where the PI_mix_ has been effectually applied.

FACS analysis of adherently growing cells requires the use of trypsin for efficient cell detachment and providing a FACS compliant suspension of solitary cells. However, trypsination is not recommended with our assay protocol as it led to higher background fluorescence that is hard to control despite extensive cell washing and application of protease inhibitors (data not shown).

### Assay comparability

**Fig 7:**
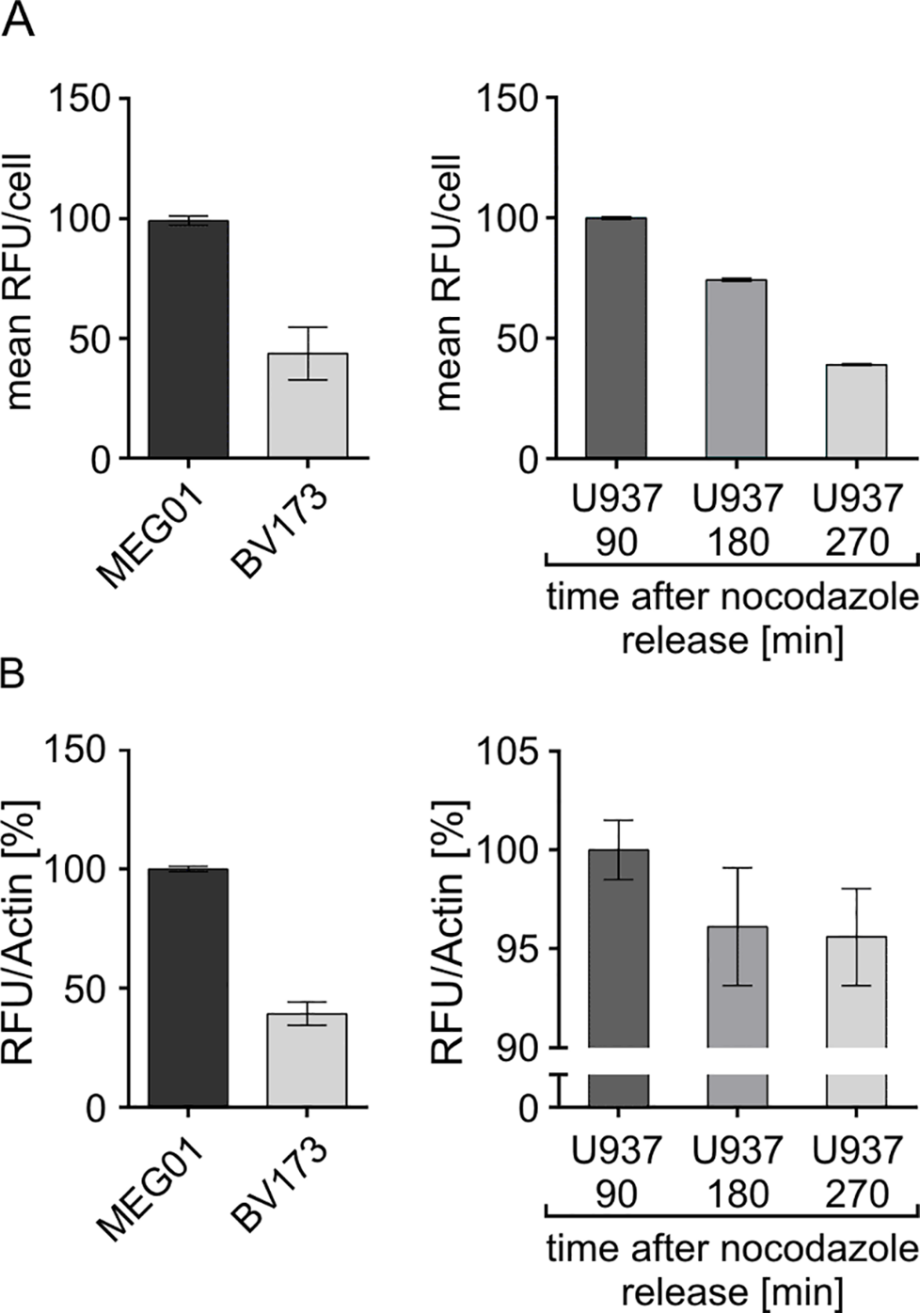
Comparability of flow cytometric- (A) and lysate-based (B) Separase activity assays. Cell lines with high (MEG01) and low (BV173) levels of Separase activity and U937 cells at distinct time points (90, 180, 270 min) after release from the nocodazole block were comparatively analyzed in parallel. For (A) the mean Rh110 fluorescence was calculated for all Separase positive cells. Data derived from the lysate-based assay (B) were calculated with respect to levels of Actin that served as internal standard (lysate loading control). Abbreviations: RFU, relative fluorescence units.

Although the whole cell extract-based Separase activity assay is quite difficult to standardize due to the lack of a reliable assay loading control (amount of protein to be estimated by Bradford assay) we have comparatively analyzed identical cell culture samples with both assays simultaneously (Fig 7). We have tested cell lines with high (MEG01) and low (BV173) average levels of Separase activity and U937 cells at distinct time-points (90, 180, 270 min) after release from the nocodazole block displaying declining levels of Separase activity (compare Fig 3A). Data derived from the lysate-based assay (Fig 7B) were calculated with respect to levels of Actin that served as internal standard after quantification by Western blot immunostaining densitometry (data not shown). Relative comparison of measured Separase activities in all three cell lines revealed overall comparability of both assays (Fig 7A vs. Fig 7B).

### Separase proteolytic activity in primary normal and leukemic cells

So far, the cell extract-based Separase assay described by Basu and coworkers allows measurement of Separase activity as a mean of all cells lysed during whole cell extract preparation. Rather it averages the Separase activity levels of all cells and does not allow for analysis of distinct cell subpopulations. [26]

It was the goal of our assay development to provide a test that allows both identifying the Separase positive cell count and monitoring the range of intercellular variation in Separase activity levels within a (tumor) cell population. This results in a Separase activity profile that comparatively describes activity in single cells. The underlying rationale was that in tumor cells the regular and tightly controlled activation of Separase may be compromised and uncoupled from cell cycle. [37] This unscheduled Separase activity in a small number of tumor cells may serve as driver of tumor heterogeneity and clonal evolution potentially contributing to the emergence of aneuploid tumor cell progeny with enhanced fidelity to escape therapeutic pressure. [27]

**Fig 8:**
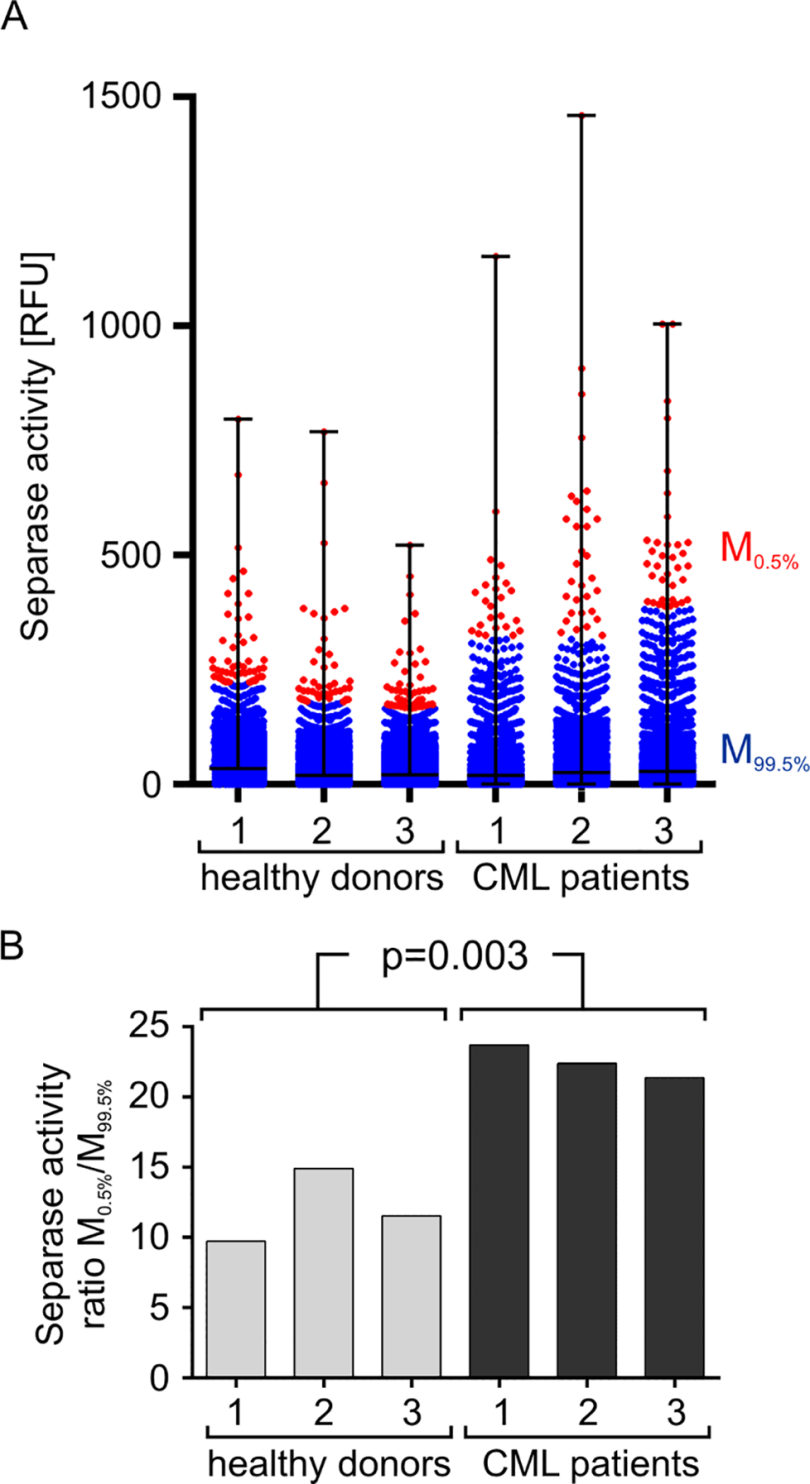
Visualization of Separase proteolytic activity on single cell level in ficollized PBMCs of healthy donors (n = 3) and CML patients (n = 3). A flow cytometry event-derived dot blot (A) represents Separase active cells ordered by their Rh110 fluorescence. The distribution of Rh110 intensities has been accentuated by coloring cells above the 99.5 percentile (= 0.5% of Separase positive cells) in red and cells below the 99.5 percentile (= 95.5% of Separase positive cells) in blue. The quotient of mean Rh110 fluorescence intensities (mean_0.5%_/mean_99.5%_) was calculated to serve as numerical value of cellular Separase activity distribution in samples under investigation (B). Abbreviations: RFU, relative fluorescence units.

For testing this concept we have comparatively analyzed the Separase proteolytic activity on single cell level in PBMCs of healthy donors (n = 3) and tyrosine kinase inhibitor treated leukemia patients with CML (n = 3) (Fig 8). The results are shown as a flow cytometric event-derived dot blot (A) that represents all Separase active cells ordered by their Rh110 fluorescence intensity (Fig 8A). It is quite obvious that the patient samples feature a higher variance in levels of Separase activity as they contain a small proportion of cells with conspicuously high Rh110 content. For a better visual estimation the distribution of Rh110 intensities was accentuated by coloring dots (= cells) above the 99.5 percentile (= 0.5% of Separase positive cells) in red and dots below the 99.5 percentile (= 95.5% of Separase positive cells) in blue. The quotient of mean Rh110 fluorescence intensities (mean_0.5%_/mean_99.5%_) was calculated to serve as numerical value of cellular Separase activity distribution in samples under investigation (Fig 8B) and to handle potential interexperimental variations in the RFU scales. Despite the small number of analyzed samples the observed differences (p = 0.003) in single cell-based distribution of Separase-associated Rh110 fluorescence between healthy and patient blood donors points to an association of increased Separase activity levels with human leukemia or the therapeutic treatment. The observed differences were not due to an increased or more variable uptake of substrate in the analyzed cells as no significant differences could be detected (p = 0.1638) when the substrate uptake ratios (M0.5%/M99.5%) of healthy donors (9.43 ± 0.64) and patients (10.56 ± 0.38) were comparatively analyzed. The observation that a small fraction of PBMCs from imatinib-treated patients samples displays increased Separase activity levels may not necessarily be due to a leukemic BCR-ABL-positive phenotype but is presumably caused by the drug itself. This is in line with our recent observation that imatinib was able to increase Separase proteolytic activity in certain leukemic cell lines. [27]

## Conclusion

In summary, we have established a novel flow cytometry-based Separase activity assay that allows detection and relative quantification of Separase proteolytic activity in single living cells. Using a standardized protocol the assay is a highly sensitive, specific, fast and user-friendly tool that allows generation of tissue- and/or tumor-specific Separase activity profiles. Especially suited for the investigaton of blood- and bone marrow-derived hematopoietic cells the resulting profiles represent “Separase fingerprints” that tells us about both the percentage of Separase positive cells and the intercellular variation of Separase activity in the analyzed cell population. Since overexpression and ectopic unscheduled activation of Separase has been considered as oncogenic promoter of aneuploidy and carcinogenesis our assay may be helpful for the prediction of chromosomal instability, clonal evolution, tumor progression and therapeutic resistance. [15,28,29,34] Recently, selective Separase inhibitors (Sepins) have been reported as potential novel therapeutics. [30] In this context we are confident that our assay will be very useful as a clinical diagnostic and prognostic tool to monitor Separase proteolytic activity in human hematological malignancies in future studies.
